# A Preliminary Inquiry Into the Potential Mechanism of Huang-Lian-Jie-Du Decoction in Treating Rheumatoid Arthritis *via* Network Pharmacology and Molecular Docking

**DOI:** 10.3389/fcell.2021.740266

**Published:** 2022-01-19

**Authors:** Chenlu Li, Jingjing Pan, Chang Xu, Zhenlin Jin, Xupeng Chen

**Affiliations:** ^1^ Department of Gastroenterology, Affiliated Yueqing Hospital, Wenzhou Medical University, Wenzhou, China; ^2^ Department of Laboratory Medicine, The First Affiliated Hospital of Wenzhou Medical University, Wenzhou, China; ^3^ Department of Intensive Care Unit, Hua Mei Hospital, University of Chinese Academy of Sciences, Ningbo, China; ^4^ Department of Hematopathology, The First Affiliated Hospital of Wenzhou Medical University, Wenzhou, China

**Keywords:** Huang-Lian-Jie-Du decoction, rheumatoid arthritis, functional enrichment analysis, network pharmacology, molecular docking

## Abstract

Huang-Lian-Jie-Du decoction (HLJDD) has been widely applied to treat inflammation-associated diseases for thousands of years in China. However, the concrete molecular mechanism of HLJDD in the treatment of rheumatoid arthritis (RA) remains unclear. In this work, network pharmacology and molecular docking were applied to preliminarily analyze the potential active ingredients, drug targets, and related pathways of HLJDD on treating RA. A total of 102 active compounds with corresponding 189 targets were identified from HLJDD, and 41 common targets were further identified by intersecting with RA-related targets. Functional enrichment analysis was performed to screen the biological pathways associated with RA. Ten hub targets were further identified through constructing the protein–protein interaction (PPI) network of common targets, which were mainly enriched in the interleukin-17 (IL-17) signaling pathway, tumor necrosis factor (TNF) signaling pathway, and Toll-like receptor signaling pathway. Furthermore, a complex botanical drugs-ingredients-hub-targets-disease network was successfully constructed. The molecular docking results exhibited that these vital ingredients of HLJDD had a stable binding to the hub targets. Among these ingredients, quercetin (MOL000098) was the most common molecule with stable binding to all the targets, and PTGS2 was considered the most important target with multiple regulations by the most active ingredients. *In vitro*, we successfully validated the inhibitory role of quercetin in the cellular proliferation of human RA fibroblast-like synoviocyte cell line (MH7A cells). These findings indicated that the potential mechanisms of HLJDD for RA treatment might be attributed to inhibiting the immune-inflammatory response, reducing the release of chemokines, and alleviating the destruction of extracellular matrix (ECM) in the synovial compartment.

## Introduction

Rheumatoid arthritis (RA) is a chronic inflammatory autoimmune disease characterized by synovitis, pannus formation, and cartilage erosion, eventually leading to progressive joint dysfunction, deformity, and increased mortality risk (Smolen et al., 2016). The prevalence rate of RA is approximately 1% in the world’s population, with higher morbidity among elderly women (Abbasi et al., 2019). So far, the treatment of RA was mainly dependent on the appropriate combination of physical, pharmacological, and surgical approaches, of which nonsteroidal anti-inflammatory drugs (NSAIDs) and disease-modifying antirheumatic drugs (DMARDs) were widely used to lighten inflammation and improve joint function (Wilsdon and Hill, 2017; Conigliaro et al., 2019). However, these drugs may result in some adverse effects, including infection, gastro-tract reaction, skin rashes, bone marrow suppression, and liver and kidney toxicity (Chen et al., 2019).

Recent advancements in pharmacy have attracted increasing attention on the use of traditional Chinese medicine (TCM) for the treatment of RA due to its significant therapeutic efficacy and mild side effects (Huang et al., 2016). As a well-known classic TCM formula for clearing heat and detoxification, Huang-Lian-Jie-Du decoction (HLJDD) is composed of *Scutellaria baicalensis* Georgi (Huangqin, HQ), *Phellodendron amurense* Rupr. (Huangbai, HB), *Coptis chinensis* Franch. (Huanglian, HL), and *Gardenia jasminoides* J.Ellis (Zhizi, ZZ) with a ratio of 2:2:3:3 (Supplementary Table S1). HLJDD has been widely applied to the treatment of inflammation-associated diseases including hepatitis (Wei et al., 2016), pneumonia (Li et al., 2021), Alzheimer’s disease (AD) (Gu et al., 2018), inflammatory bowel disease (IBD) (Yuan et al., 2019), and RA (Hu et al., 2013). Furthermore, numerous studies have shown that HLJDD could significantly reduce the levels of inflammatory cytokines such as TNF-α and inflammatory mediators such as prostaglandin E2 (PGE2) to realize the anti-inflammatory efficacy (Fang et al., 2004; Lu et al., 2011). *In vitro*, Sung et al. (2012) demonstrated that quercetin could significantly inhibit unstimulated and IL-1β induced proliferation of rheumatoid synovial fibroblasts (RASFs) and the messenger ribonucleic acid and protein expression of MMP-1, 3, COX-2. In animal models, Hu et al. also successfully demonstrated the treatments of HLJDT on collagen-induced arthritis in rats (Hu et al., 2013). During the period of COVID-19, HLJDD was also found to play a therapeutic role in COVID-19 through regulating multiple signaling pathways based on targeting genes such as IL6, IL10, MMP9, NOS2, VEGF, and TGFβ1 (Liu et al., 2021). Nevertheless, the concrete molecular mechanism of HLJDD in the treatment of RA was still unclear.

As a novel, promising, and cost-effective drug research approach based on bioinformatics and pharmacology, network pharmacology has been widely used in studies on the molecular mechanism of drugs with molecular docking. In previous studies, Yang et al. (2013) identified berberine, baicalin, and geniposide as three major ingredients of HLJDD and further demonstrated their anti-inflammatory effects through inhibiting NF-κB and MAPK signaling pathways in a dose-dependent manner. In addition, using network pharmacology and metabolomics analysis, Qu et al. found the antidepressant effects of HLJDD through candicine, berberine, tetrahydroberberine, and baicalein to regulate SLC6A4 and MAOA in tryptophan metabolism (Qu et al., 2021). In Li’s study, the network pharmacology and molecular docking method was also well conducted to decode the potential mechanism of HLJDD in treating pneumonia and construct a complex botanical drugs-ingredients-targets-disease network (Li et al., 2021). These studies provided a convincing possibility that network pharmacology and molecular docking analysis can be effectively applied to investigate potential therapeutic targets of HLJDD and facilitate a deep understanding of its underlying mechanism in RA.

**FIGURE 1:**
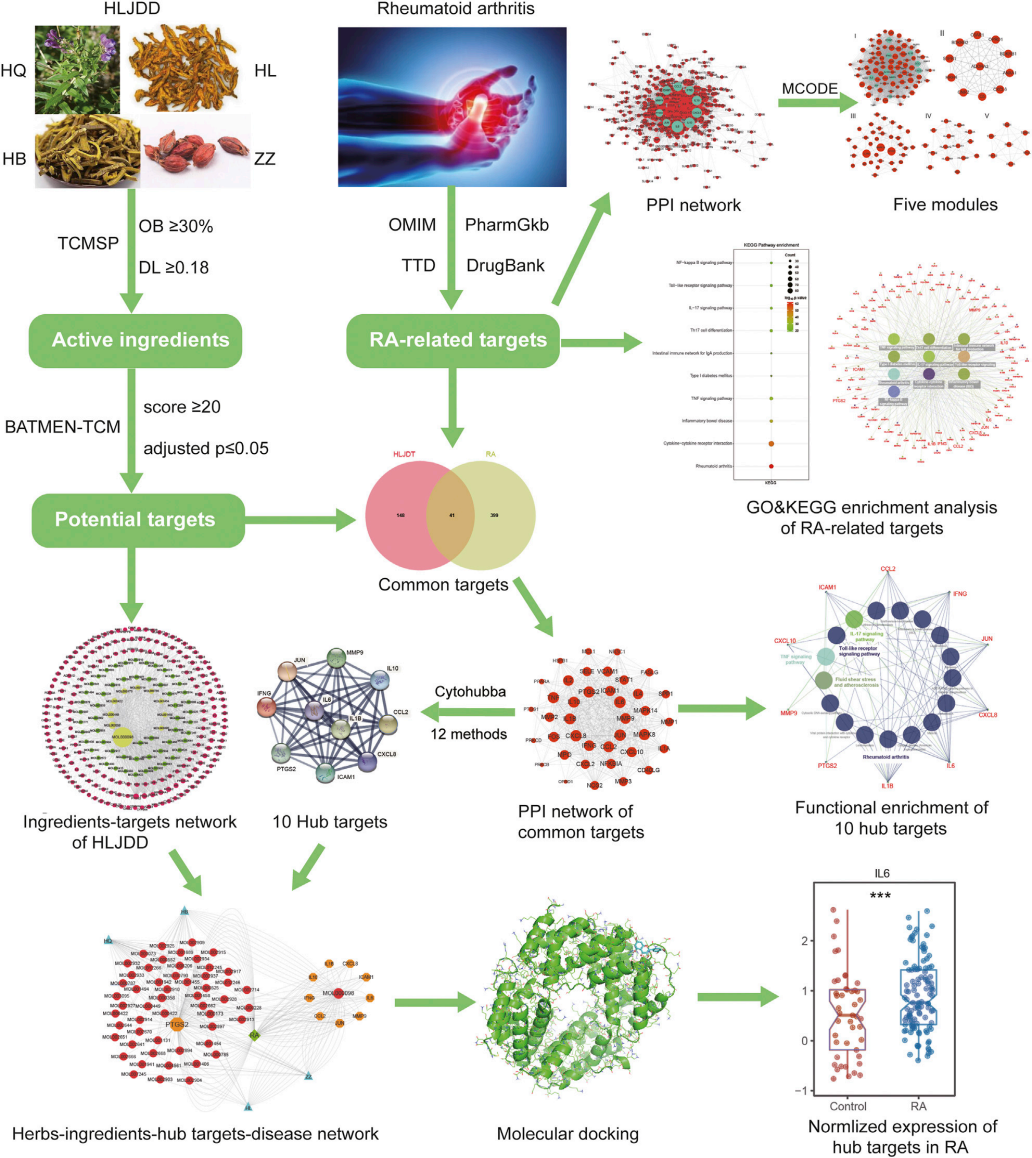
The summary and description of the study workflow in the potential mechanism of HLJDD in treating RA. The active ingredients and corresponding targets of HLJDD were obtained through TCMSP and BATMEN-TCM database. The RA-related targets were downloaded from four different databases, and the common targets were identified by intersecting ingredient targets and RA-related targets. GO and KEGG enrichment analysis was conducted, and PPI network with cytoHubba plug-in was used to select hub targets in the common targets. Finally, the complex botanical drugs-ingredients-hub-targets-disease network was constructed and validated by molecular docking and normalized expression of RA-related target genes from GEO datasets.

In the present study, we investigated the potential targets and pathways of HLJDD in treating RA and constructed the botanical drugs-ingredients-targets-disease network through network pharmacology analysis and molecular docking validation. We were convinced that our results would help in illuminating HLJDD’s possible mechanism of the treatment in RA, thus improving the curative effect and prognosis for RA. The workflow of the whole process of our study is shown in Figure 1.

## Materials and Methods

### Screening Active Ingredients and Potential Targets in HLJDD

In this study, the Traditional Chinese Medicine Systems Pharmacology (TCMSP) (Ru et al., 2014) database was utilized to screen active ingredients of HLJDD by using “Huangbai,” “Huanglian,” “Huangqin,” and “Zhizi” as keywords with the filtration criteria of bioavailability (OB) ≥30% and drug-likeness (DL) ≥0.18, respectively (Song et al., 2018). The corresponding molecular structure, structural information, and PubChem CID of those active ingredients were further obtained from the “PubChem” online tool (Wang et al., 2017). Subsequently, using the above structural information of ingredients, we utilized the “BATMEN-TCM” online tools to identify potential targets in HLJDD with a score ≥20 and adjusted *p* ≤ 0.05 (Liu et al., 2016).

### Identification of Target Genes in Rheumatoid Arthritis

The RA-associated target genes were obtained from the OMIM database (Rigden and Fernandez, 2020), PharmGKB database (Whirl-Carrillo et al., 2012), DrugBank database (Wishart et al., 2018), and TTD database (Li Y. H. et al., 2018), respectively. By searching “rheumatoid arthritis” as the keyword, we identified 12, 13, 299, and 157 targets related to RA from these databases, respectively. The UniProt database was further employed to standardize their protein structures in unique ways (UniProt Consortium, 2019).

### Construction of PPI Network and Cluster Analysis

The PPI data of the potential target interactions were downloaded from the STRING database (version 11.0) (Szklarczyk et al., 2019) with setting the Organism as “*Homo sapiens*” and confidence score ≥0.4. Then, the PPI network was constructed and visualized by the Cytoscape software (Shannon et al., 2003), and hub targets were detected according to levers of degree value (the number of interactions for each node) in the PPI network. In the further cluster analysis, the plug-in MCODE (Deng et al., 2019) was used to identify significant modules using the filter conditions: k core = 2, maximum depth = 100, node score cutoff = 0.2, and degree cutoff = 2.

### GO and KEGG Functional Enrichment Analysis

To investigate the biological function of potential targets in RA, we performed gene ontology (GO) analysis, including biological processes (BP), molecular functions (MF), and cellular components (CC), and Kyoto Encyclopedia of Genes and Genomes (KEGG) functional enrichment analysis. These analyses were conducted through the online tool “g:Profiler” (Raudvere et al., 2019) with Bonferroni correction and adjusted *p* ≤ 0:05. Moreover, the ClueGo (Bindea et al., 2009) and CluePedia (Bindea et al., 2013) plug-in of Cytoscape software were further applied to visualize the network of significant pathways and their corresponding target genes.

### Identification of Hub Targets and Critical Network of HLJDD on RA

The common targets between HLJDD and RA were identified through taking the intersection, and the PPI network of these targets was also constructed and visualized in the Cytoscape software. Subsequently, we used plug-in cytoHubba (Chin et al., 2014) to explore important nodes in the above PPI network by 12 topological algorithms including MCC, MNC, DMNC, BottleNeck, EcCentricity, EPC, Radiality, Betweenness, Closeness, Clustering Coefficient, Degree, and Stress. The top 10 nodes identified by each topological algorithm were chosen to identify the shared genes more than six ways as the most pivotal hub genes in the network (Luo et al., 2020). Finally, a complex botanical drugs-ingredients-hub-targets-disease network was further constructed and visualized in the Cytoscape.

### Visualization and Validation of Molecular Docking

To validate the binding of hub targets and active ingredients in HLJDD, the 3D molecular conformations of ingredients were retrieved from the PubChem Compound database. The crystal structures of target proteins were obtained from the RCSB Protein Data Bank (PDB) database (Burley et al., 2021). AutoDock 4.2.6 tool was used to remove the redundant structure, ligands, and water molecules and add polar hydrogen atoms and partial charges into protein crystal structures before running molecular docking (Li H. et al., 2018). The visualization of molecular docking was exhibited by PyMol software (Seeliger and de Groot, 2010). The transcriptomic dataset of peripheral blood mononuclear cells (PBMCs) (GSE17755, including 112 RA and 45 controls) was downloaded from Gene Expression.

Omnibus (GEO) database to validate the normalized expression of RA-related target genes. Values were presented as the mean ± standard deviation (SD), and the comparison between groups was performed using the Wilcoxon test by the “ggpubr” R package. *p* < 0.05 was considered statistically significant.

### Cell Culture and Treatment

Human RA fibroblast-like synoviocyte cell line (MH7A cells) was purchased from the Riken cell bank (Ibaraki, Japan), and cells were maintained in DMEM supplemented with 10% fetal bovine serum (FBS) and 1% penicillin-streptomycin solution (HyClone, Shanghai). Subsequently, the MH7A cells were plated into a six-well plate (5 × 10^3^ cells/well) and treated with different concentrations of quercetin (0, 25, 50, 75, and 100 μM; Sigma, St Louis, MO, United States) in 37°C incubator with 5% CO^2^ atmosphere for 24 h. Meanwhile, the culture medium with 10% DMEM and 0.05% DMSO but without MH7A cells was set as control groups and also plated in the same condition for 24 h.

### Cell Proliferation Assay

Equal numbers of the above MH7A cells were plated in a 96-well plate (5 × 10^3^ cells/well), and Cell Counting Kit-8 (CCK-8) (Dojindo, Tokyo, Japan) (10 μL/well) was applied to measure the cell proliferation at 24 h. Finally, the imaging of cell proliferation was recorded, and the absorbance value (OD value; A_450nm_) of MH7A cells was further detected using a Microplate Reader (Thermo Fisher, MA). Based on the OD value of different quercetin concentrations, we successfully drew the drug-concentration curve to identify the half-maximal inhibitory concentration (IC50) value of quercetin in RA.

### Statistical Analysis

All statistical analyses were performed in R software (version 4.0.1, https://www.r-project.org/). Continuous variables were presented as mean ± standard deviation (SD), and Wilcoxon signed-rank test was used to compare continuous variables. The two-tailed *P* value <0.05 was considered statistically significant.

## Result

### Identification of Potential Targets-Active Ingredients Network

**FIGURE 2:**
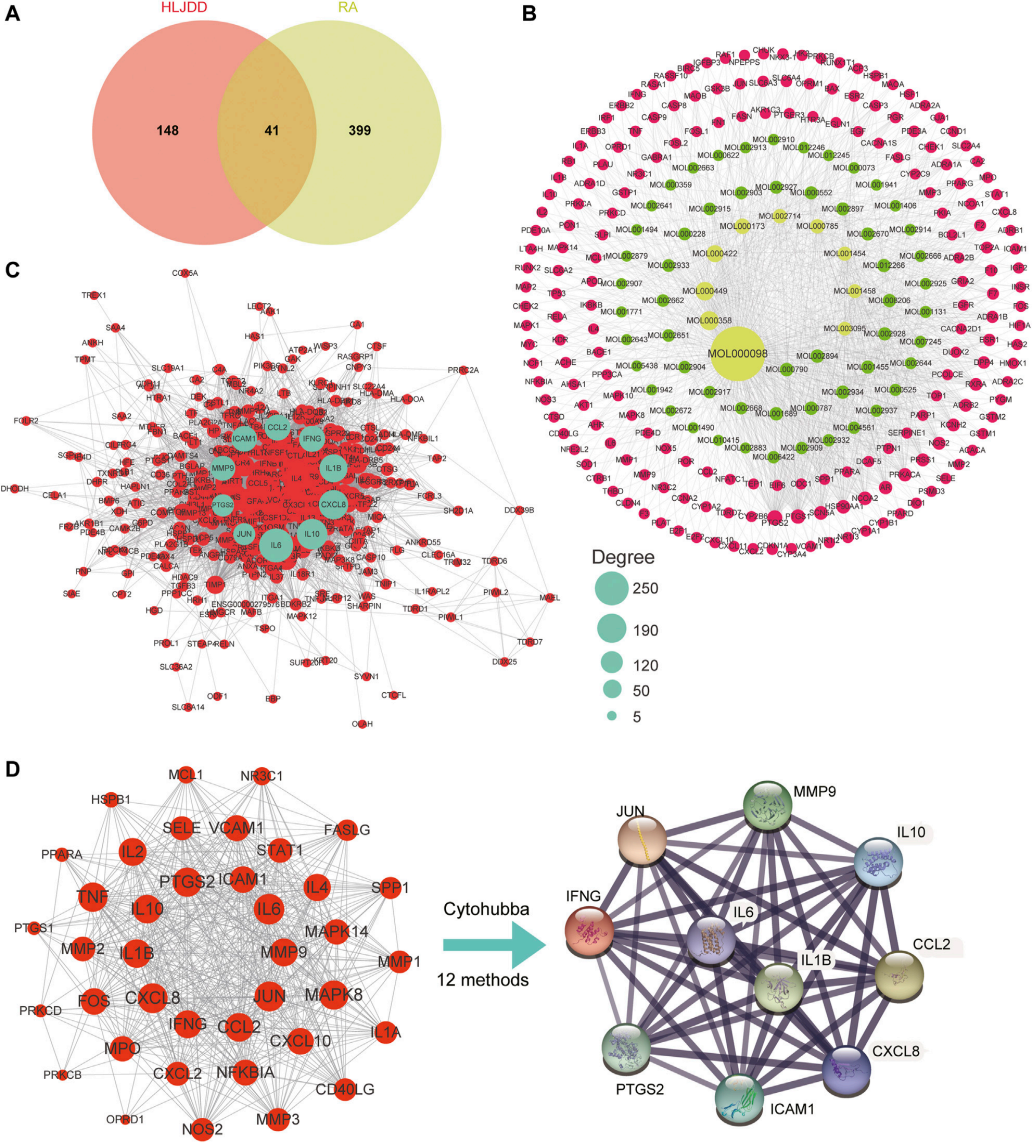
Identification of potential targets-active ingredients network. **(A)** Venn diagram of the common targets of active ingredients and RA-related targets. **(B)** The active ingredients-targets network of HLJDD. **(C)** The PPI network of all RA-related targets. The cyan nodes represent the 10 hub targets obtained from the subsequent cytoHubba analysis. **(D)** The PPI network of 39 common targets between RA-related and HLJDD; The PPI networks of 10 vital targets were identified using Cytohubba plug-in with approval of more than 6 methods.

Based on the criteria of OB ≥ 30% and DL ≥ 0.18, a total of 102 active ingredients were predicted in HLJDD through the TCMSP database, including 37 from HB, 14 from HL, 36 from HQ, and 15 from ZZ (Supplementary Figure 1A). Moreover, we also screened a total of 1008 corresponding targets of HLJDD using the TCMSP and BATMEN-TCM, of which 271 were from HB, 216 from HL, 226 from HQ, and 295 from ZZ (Supplementary Table S3). Combined with the 102 active ingredients, 189 targets of these ingredients were further identified, and the complex targets-active ingredients network is constructed in Figure 2A. Notably, by setting the degree value ≥ 45, the top 10 active ingredients were identified in the network, including quercetin (MOL000098), beta-sitosterol (MOL000358), stigmasterol (MOL000449), kaempferol (MOL000422), wogonin (MOL000173), baicalein (MOL002714), palmatine (MOL000785), berberine (MOL001454), coptisine (MOL001458), and 5-hydroxy-7-methoxy-2-(3,4,5-trimethoxyphenyl) chromone (MOL003095). The detailed characteristics of these active ingredients and targets are shown in Supplementary Table S2 and Supplementary Table S3. To further identify the changes of gene expression in the progress of RA, four databases, namely, OMIM, GenBank, PharmGKB, and TTD, were screened to obtain RA-related target genes, and a total of 440 targets of RA were obtained (Supplementary Figure 1B, Supplementary Table S4). Subsequently, comparing the target genes of HLJDD and RA, we found that RA shared 41 common targets with that of active ingredients (Figure 2A).

### PPI Network and Cluster Analysis of Disease Targets in RA

To identify central target genes of RA in the physical interaction network and provide potential clues for the possible pathogenic mechanisms of RA, we successfully constructed an interconnected PPI network of 440 RA-related targets with 394 nodes and 9391 edges (Figure 2C) based on the STRING database. Using the MCODE plug-in in Cytoscape with MCODE score greater than 5, we further identified five clusters with close interconnection (Supplementary Figure 1C, Table 1). The blue nodes represented the significant targets with high degree values in the network, which might play essential roles in the progression of RA.

**TABLE 1:** Cluster information of RA protein–protein interaction (PPI) network.

Cluster	Score	Nodes	Edges	Gene symbol
I	70.51	87	3032	CRP, CXCL12, SELL, FASLG, CCL2, MMP2, CXCL2, TIMP1, CXCR3, TNFSF13B, TNFRSF1A, CSF2, CX3CL1, CD44, CD19, JUN, VCAM1, CXCL8, CCL3, ITGAM, IL10RA, FOXP3, ALB, IL33, TNFSF11, VEGFA, CCL20, CXCR4, INS, CXCL1, FCGR2B, CXCL10, IL17A, ANXA5, CD86, FCGR2A, CCL5, CCR1, CSF1, SELE, STAT3, CXCL5, IL13, CD80, IL7, CCR5, CCR2, CSF1R, CCRL2, CD28, CXCR5, CD40, TLR9, TLR2, NLRP3, IL4, MAPK1, STAT1, IL2RA, CTLA4, TNF, IL6, NOS2, MAPK8, CD40LG, CASP1, KLRK1, IFNG, MMP9, CD69, IL2, TLR1, CSF3, PTGS2, TNFRSF1B, GZMB, GPR29, IL15, IL6R, CX3CR1, IL18, TLR4, IL10, IL1B, IL1A, ICAM1, IL1R1
II	11.00	11	55	CNR2, HRH4, OPRD1, S1PR1, CXCL6, BDKRB2, C5, BDKRB1, C5AR1, ANXA1, ADORA3
III	9.71	35	165	LTA, ITGAL, IRAK1, MAPK14, TGFB1, NFKB1, IL22, IL17RA, IL23A, NOD2, REL, HMGB1, SPP1, FAS, RELB, ITGB2, ITGA4, IL16, MMP1, MMP3, MIF, NFKBIA, SYK, IL23R, FOS, IL2RB, STAT4, IL21, ELANE, IL11, PDCD1, MPO, CD4, IL1RN, IFNB1
IV	8.86	15	62	HLA-DOA, PTPN22, HLA-DPB1, HLA-B, NLRP1, HLA-DMA, HLA-DRB5, MICA, HLA-DMB, HLA-DQB1, HLA-DQA2, CD247, CTSL, HLA-DQA1, P2RX7
V	7.00	7	21	MAEL, TDRD6, TDRD1, PIWIL1, DDX25, PIWIL2, TDRD7

### Functional Enrichment Analysis of Disease Targets in RA

**FIGURE 3:**
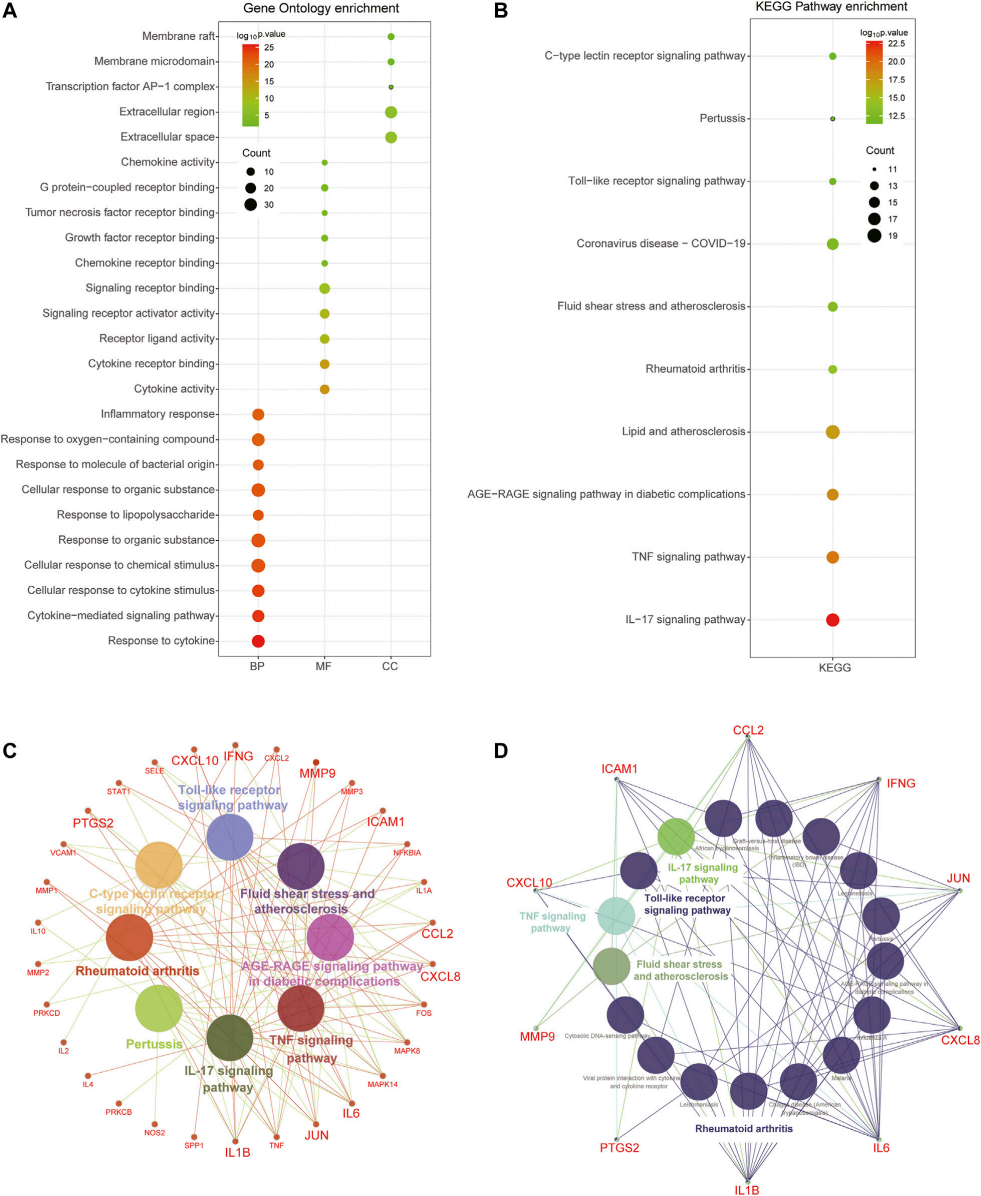
Functional enrichment analysis of total RA-related targets. **(A)** Bubble diagram showing the results of GO enrichment analysis, including top 10 terms in BP, MF, and CC, respectively. **(B)** Top 10 pathways of KEGG enrichment analysis were associated with immune activation. **(C)** The network showing the detailed genes involved in the top 10 pathways and the hub targets were enlarged. **(D)** The network showing the KEGG pathways enrichment of 10 hub targets.

In order to further interpret biological processes associated with the RA-related targets, GO and KEGG enrichment were performed based on the 440 target genes. It turned out that these target genes were enriched in 1237 BPs, 81 MFs, 67 CCs, and 76 KEGG pathways (Supplementary Table S5). The results showed that the biological processes of RA were mainly related to the immune response process, such as “immune system process,” “response to organic substance,” “cellular response to cytokine stimulus,” and “inflammatory response.” The molecular functions of RA might be related to “signaling receptor binding,” “molecular transducer activity,” and “signaling receptor activity.” The cellular components were principally associated with “cell periphery,” “extracellular region,” and “plasma membrane” (Figure 3A). The KEGG pathway analysis also revealed these targets were significantly enriched in immune-activated associated diseases and pathways, including “rheumatoid arthritis,” “inflammatory bowel disease,” “cytokine-cytokine receptor interaction,” “TNF signaling pathway,” “NF-kappa B signaling pathway,” and “Th17 cell differentiation” (Figure 3B). In addition, the concrete network of the KEGG pathways indicated that massive inflammatory cytokines participated in the progression of RA (Figure 3C).

### Identification of Hub Genes and Critical Network of HLJDD on RA

In order to identify the hub genes of HLJDD in treating RA, we also imported the 41 common targets into the STRING database and subsequently constructed their PPI network (Figure 2D). The cytoHubba plug-in was further applied to calculate the significance of targets with 12 methods, and we found that there were 10 hub genes shared with more than 6 topological analyses (MMP9, IL1β, IFNG, IL10, ICAM1, CCL2, PTGS2, IL6, CXCL8, and JUN) by selecting top 10 genes of each method (Table 2). The PPI network of the hub genes included 10 nodes and 45 edges, with an average node degree of 9 and *P* value of 3.22e-14 (Figure 2D).

**TABLE2:** The top 10 genes of 12 methods of cytoHubba plug-ins in Cytoscape software.

DMNC	MNC	MCC	Degree	EPC	BottleNeck	EcCentricity	Closeness	Radiality	Betweenness	Stress	Clustering Coefficient
MMP3	MMP3	MMP3	MMP9	IFNG	MMP9	MMP9	MMP9	MMP9	MMP9	MMP9	MMP9
MMP1	MMP1	MMP1	IL1B	JUN	IL1B	IL1B	IL1B	IL1B	IL1B	IL1B	IL1B
SELE	SELE	SELE	IFNG	CXCL8	IFNG	IFNG	IFNG	IFNG	IFNG	IFNG	IFNG
ICAM1	ICAM1	ICAM1	IL10	IL10	IL10	IL10	IL10	IL10	IL10	IL10	IL10
IL1A	IL1A	IL1A	ICAM1	CCL2	ICAM1	ICAM1	ICAM1	ICAM1	ICAM1	ICAM1	ICAM1
VCAM1	VCAM1	VCAM1	CCL2	MMP9	CCL2	CCL2	CCL2	CCL2	CCL2	CCL2	CCL2
MMP9	MMP9	MMP9	PTGS2	IL1B	PTGS2	PTGS2	PTGS2	PTGS2	PTGS2	PTGS2	PTGS2
CXCL2	CXCL2	CXCL2	IL6	PTGS2	IL6	IL6	IL6	IL6	IL6	IL6	IL6
IL4	IL4	IL4	CXCL8	IL6	CXCL8	CXCL8	CXCL8	CXCL8	CXCL8	CXCL8	CXCL8
CXCL10	CXCL10	CXCL10	JUN	ICAM1	JUN	JUN	JUN	JUN	JUN	JUN	JUN

Furthermore, by selecting the hub gene-related active ingredients in HLJDD, we successfully constructed the botanical drugs-ingredients-targets-disease network (Figure 3D), containing 1 disease, 4 botanical drugs, 54 ingredients, and 10 hub genes. It could be seen that quercetin (MOL000098), beta-sitosterol (MOL000358), wogonin (MOL000173), kaempferol (MOL000422), coptisine (MOL001458), and stigmasterol (MOL000449) were pivotal ingredients that were identified with high degree value, suggesting these ingredients might be the material basis for HLJDD in treating RA.

### Selecting and Analyzing Critical GO and KEGG of Hub Genes

**FIGURE 4:**
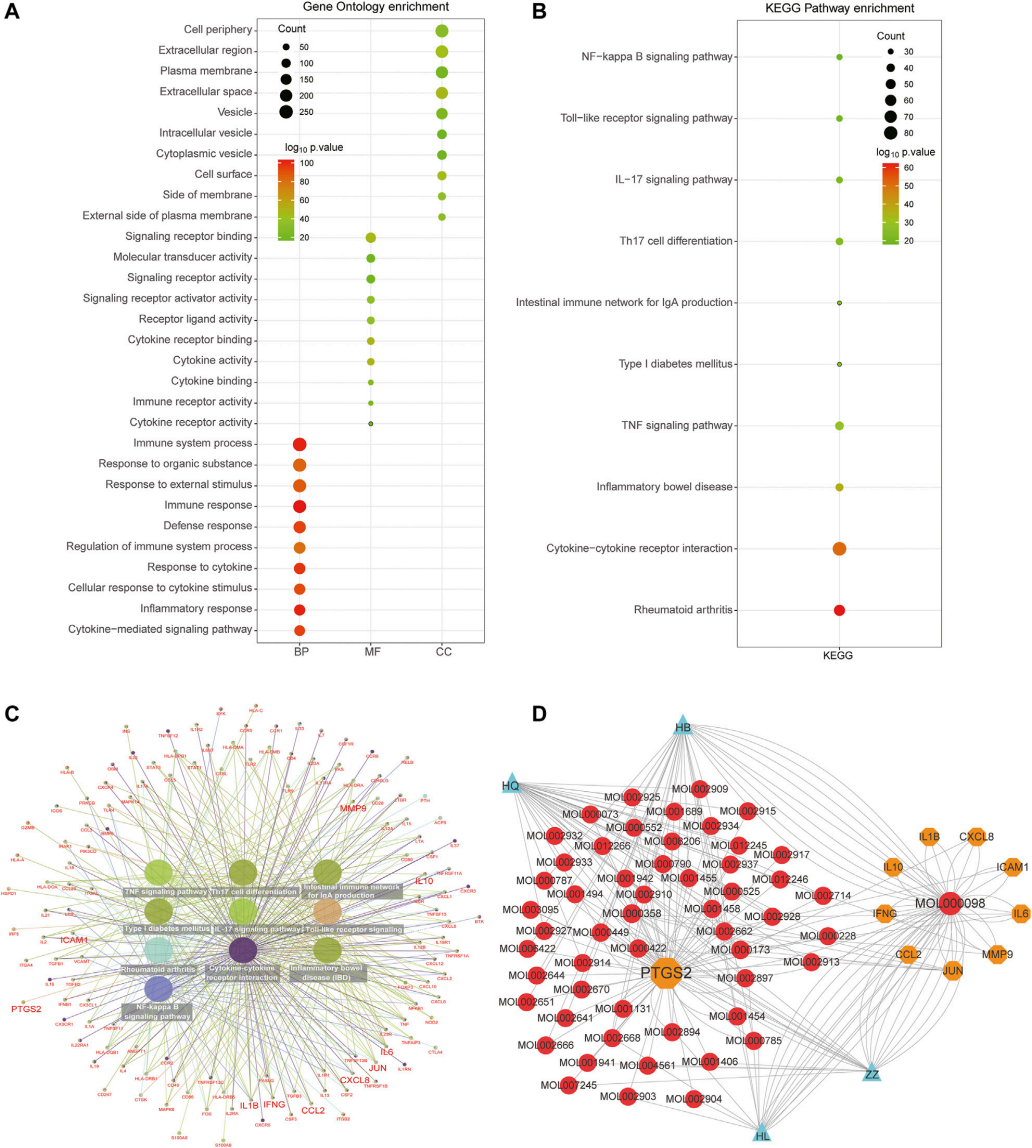
Functional enrichment analysis of 41 common and 10 hub targets. **(A)** Top 10 terms of BP, MF, and CC using GO enrichment analysis of 41 common targets. (B) Top 10 KEGG pathways of 41 common targets. **(C)** The network included detailed targets of these KEGG pathways. **(D)** The complex botanical drugs-ingredients-hub-targets-disease network of HLJDD in the treatment of RA. The blue nodes represent botanical drugs. The red nodes represent botanical drugs activation ingredients. The orange nodes represent hub targets. The green node represents RA disease.

In order to further interpret the mechanism of HLJDD in the treatment of RA, we used the common 41 targets to perform GO and KEGG functional enrichment analysis. A total of 631 BPs, 5 CCs, 19 MFs, and 78 KEGG pathways were identified, and the top 10 biological processes are exhibited in Figure 4. GO enrichment analysis demonstrated that the common targets were mainly enriched in the inflammatory response and cytokine-associated processes such as “inflammatory response,” “cellular response to cytokine stimulus,” “cytokine-mediated signaling pathway,” and “response to cytokine.” The main cellular components were located in the extracellular region, and the molecular functions were mainly enriched in “signaling receptor binding,” “cytokine receptor binding,” and “cytokine activity” (Figure 4A). As to KEGG pathways analysis, the most significant pathways of the common targets were “IL-17 signaling pathway,” “TNF signaling pathway,” “AGE−RAGE signaling pathway in diabetic complications,” and “rheumatoid arthritis” (Figure 4B), and the interaction network also demonstrated that the ten hub genes participated in these significant pathways (Figure 4C). Moreover, KEGG analysis indicated that the hub targets were significantly enriched in biological processes associated with immune activation, including IL-17 signaling pathway, Toll-like receptor signaling pathway, TNF signaling pathway, fluid shear stress, atherosclerosis, and rheumatoid arthritis pathways (Figure 4D).

### Validation of Hub Genes by Molecular Docking and mRNA Expression

**FIGURE 5:**
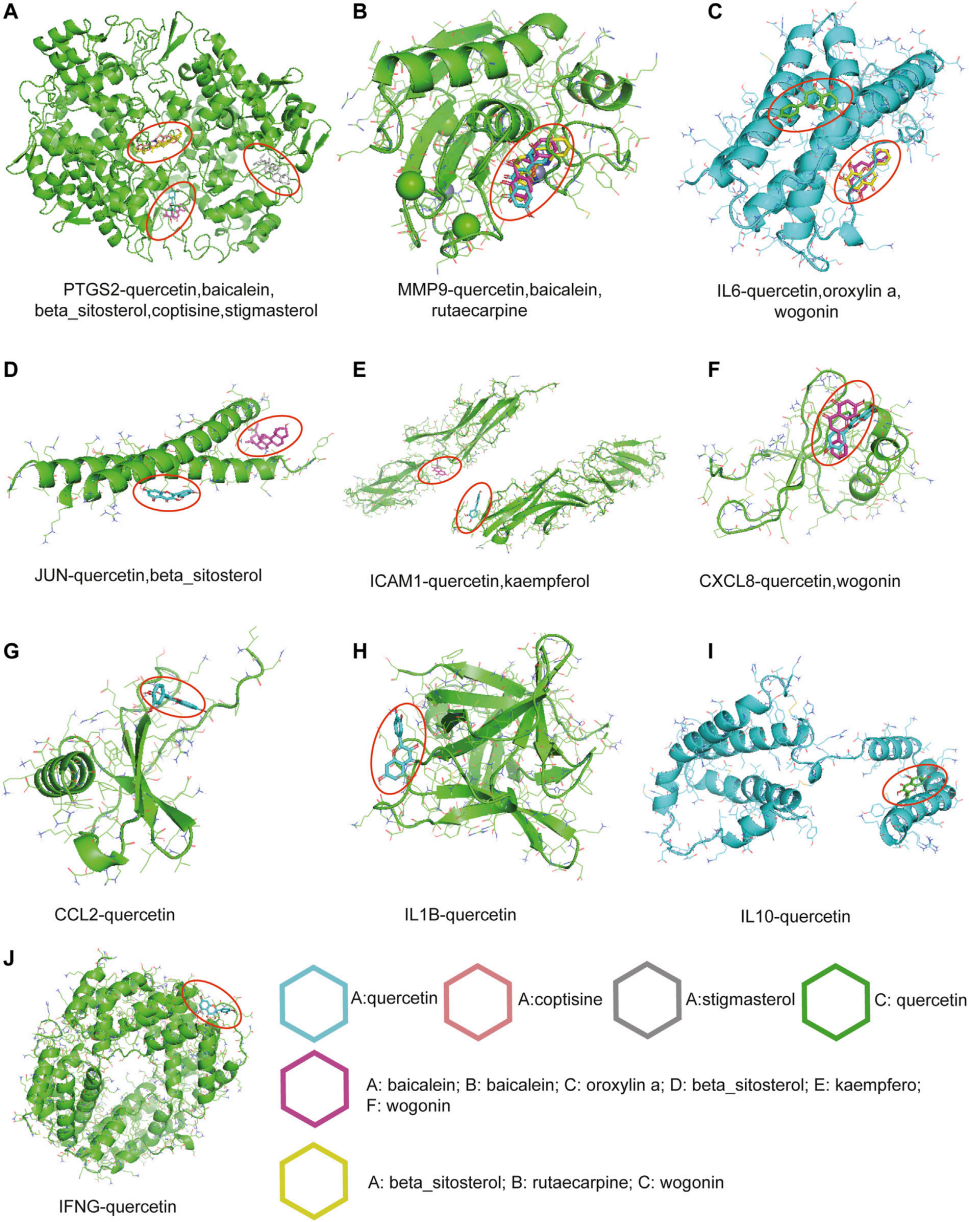
The molecular docking results between active ingredients and hub targets. **(A)** PTGS2, **(B)** MMP9, **(C)** IL6, **(D)** JUN, **(E)** ICAM1, **(F)** CXCL8, **(G)** CCL2, **(H)** IL1β, **(I)** IL10, **(J)** IFNG. Different molecular rings with different colors represent different active ingredients.

**FIGURE 6:**
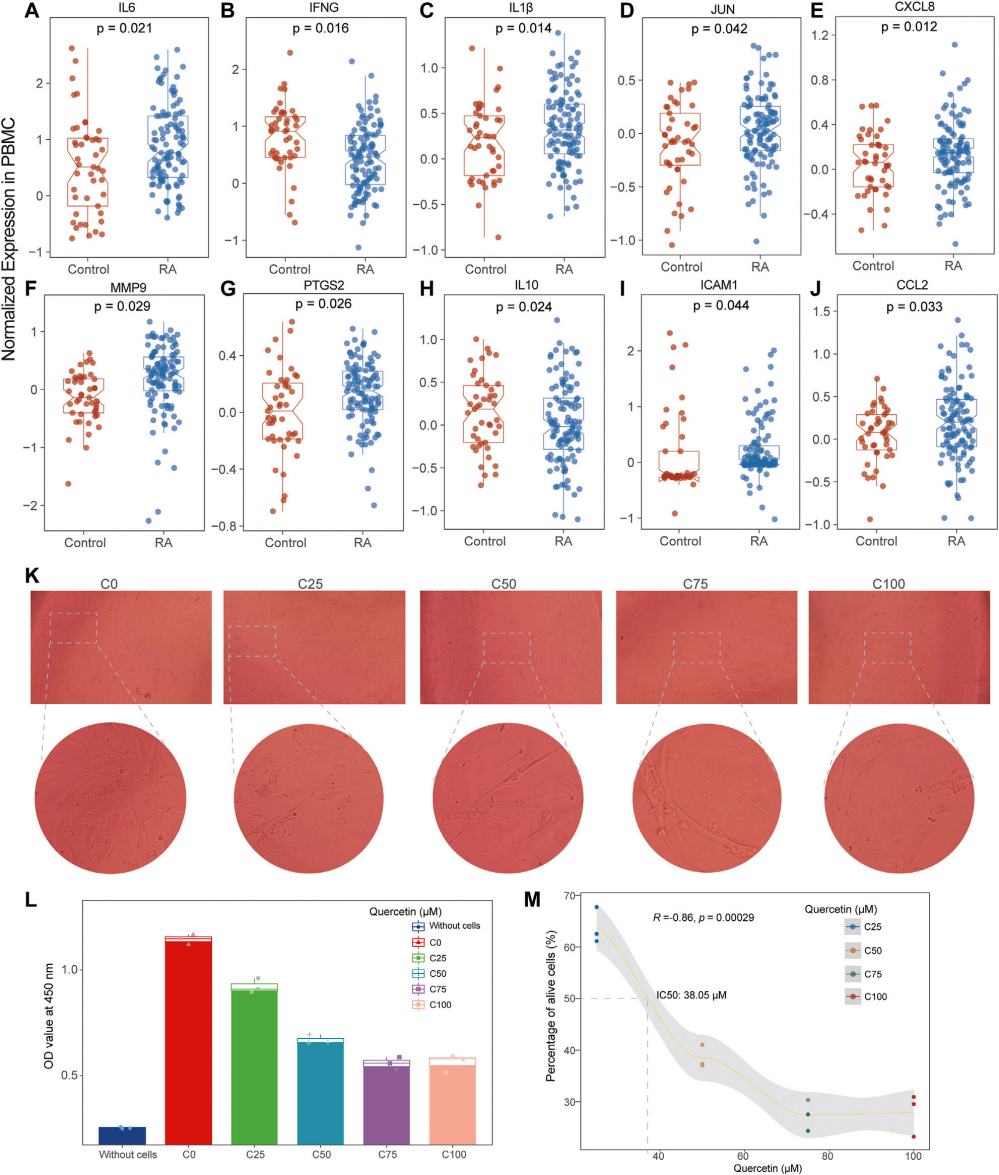
Normalized expression of 10 hub genes in PBMC of RA and control and cell proliferation assay. **(A–J)** The results showed that the expression levels of IL6, IL1β, CXCL8, MMP9, PTGS2, ICAM1, and CCL2 were increased in RA while the expression levels of IFNG and IL10 were decreased in RA compared with the control. **(K,L)** The histogram and cellular imaging showed the quercetin’s inhibitory effectiveness was generally increased with the enhancement of quercetin. **(M)**. The drug-concentration curve displayed the IC50 value of quercetin was identified as 38.02 μM in the treatment of RA.

To further evaluate and validate the effective binding of targets and active ingredients, ten hub genes and nine pivotal ingredients were used to perform molecular docking as receptors and ligands, respectively. The stability and strength of the binding were based on the binding energy. The lower the binding energy value was, the more stable the binding was. Setting the binding energy value < −5.0 kcal/mol as the threshold, we successfully demonstrated the ligands binding well to the target receptor with the lowest energy (Table 3). Among these ingredients, quercetin (MOL000098) was the most significant molecule with stable binding to all the targets, and PTGS2 was considered the most important target with multiple regulations by the most active ingredients (Figure 5). Normalized expression of 10 hub targets is also validated in GEO datasets, and it revealed that IL6, IL1β, JUN, CXCL8, MMP9, PTGS2, ICAM1, and CCL2 were significantly upregulated. In contrast, IFNG and IL10 were significantly downregulated in the PBMC of RA patients compared to controls (Figures 6A–J).

**TABLE 3:** Molecular docking results between ligands and core target receptors.

Target genes	PDB ID	Active ingredients	Binding energy (kcal/mol)
PTGS2	5F19	Quercetin	-9.6
PTGS2	5F19	Baicalein	-9.3
PTGS2	5F19	Beta-sitosterol	-9.7
PTGS2	5F19	Coptisine	-9.6
PTGS2	5F19	Stigmasterol	-9.0
IL6	1IL6	Quercetin	-7.4
IL6	1IL6	Wogonin	-7.2
IL6	1IL6	Oroxylin A	-7.0
MMP9	4H3X	Quercetin	-10.7
MMP9	4H3X	Baicalein	-10.1
MMP9	4H3X	Rutaecarpine	-8.6
CXCL8	4XDX	Quercetin	-6.8
CXCL8	4XDX	Wogonin	-6.7
JUN	1JUN	Quercetin	-5.5
JUN	1JUN	Beta-sitosterol	-5.5
ICAM1	1D3L	Quercetin	-6.2
ICAM1	1D3L	Kaempferol	-5.9
IFNG	1HIG	Quercetin	-7.7
IL1β	31BI	Quercetin	-7.5
IL10	1ILK	Quercetin	-6.7
CCL2	1DOM	Quercetin	-6.1

### Quercetin Inhibited Cell Proliferation in MH7A Cells

Initially, we estimated the proliferation of MH7A cells after the treatment of series quercetin concentrations (0, 25, 50, 75, and 100 μM) and found the inhibitory effectiveness was generally increased with the enhancement of quercetin (Figure 6K). However, the growth did not increase speedily when the concentration was more than 50 μM (Figure 6L), and the IC50 value of quercetin was identified as 38.02 μM according to the drug-concentration curve (Figure 6M).

## Discussion

As one of the most common systematic autoimmune disorders, RA has a high proportion of incidence and joint deformity with high disease activity. HLJDD is a classical formula from TCM and has been widely used to treat inflammation-related diseases for thousands of years, and recent preclinical studies also demonstrated its effective treatment in RA (Hu et al., 2013). However, its concrete mechanism of the clinical effects remains unclear. Therefore, we investigated the potential mechanisms of the therapeutic effect of HLJDD in RA employing network pharmacology and molecular docking analysis.

In our study, the ingredients-target network of HLJDD was constructed using 102 compounds, and their 189 targets and complicated network demonstrated that most ingredients of the HLJDD could regulate multiple targets. Combined with the PPI network analysis, nine pivotal ingredients were ultimately identified, including quercetin, coptisine, stigmasterol, baicalein, oroxylin A, beta-sitosterol, kaempferol, wogonin, and rutaecarpine. Interestingly, using High Performance Liquid Chromatography (HPLC) analysis, Yuan et al. (2019) detected the contents of 13 ingredients and indirectly supported our findings, including phellodendrine (1), chlorogenic acid (2), magnoflorine (3), geniposide (4), coptisine (5), epiberberine (6), jatrorrhizine (7), berberine (8), palmatine (9), baicalin (10), wogonoside (11), wogonin (12), and oroxylin A (13). Quercetin was a class of flavonol that has unique therapeutic properties, including anti-inflammatory, antioxidant, anticarcinogenic, and antiviral activities (Li et al., 2016). Wogonin was a natural flavonoid derived from the root extract of Huangqin and had demonstrated potential anti-inflammatory and chondroprotective effects in osteoarthritis through activating the ROS/ERK/Nrf2 signaling pathway (Khan et al., 2017). Baicalein was considered an anticarcinogenic compound to inhibit the deterioration of multiple tumors, such as hepatocellular carcinoma and breast cancer (Bie et al., 2017; Yan et al., 2018). Kaempferol has been reported as a potent immunosuppressant that significantly reduced the hypernomic immune response, including autoimmune and chronic inflammation responses (Lin et al., 2011). In this study, the above compounds regulated most RA-related targets and exhibited anti-inflammatory effects. Furthermore, the molecular docking results exhibited appropriate docking between the crystal structures of target proteins and activated ingredients. Increasing evidence indicated quercetin could inhibit cell apoptosis and reduce the expression levels of inflammatory factors such as IL6 and TNF-α (Wang et al., 2019). In addition, quercetin significantly inhibited MMP9’s expression through NF-kappa B signaling pathway to attenuate cell migration and invasion (Lu et al., 2018). Interestingly, Sung et al. (2012) also demonstrated that quercetin could significantly inhibit unstimulated and IL-1β induced proliferation of rheumatoid synovial fibroblasts (RASFs) and the messenger ribonucleic acid and protein expression of MMP-1, 3, COX-2. Consistent with previous literature, through cell experiments, we successfully validated the inhibitory role of quercetin in the cellular proliferation of MH7A cells *in vitro*. These results suggested that the therapeutic mechanism of activated ingredients of HLJDD focused on anti-inflammatory and anticarcinogenic biological effects, interpreting the efficacious treatment in RA.

Moreover, 10 hub targets (MMP9, IL1β, IFNG, IL10, ICAM1, CCL2, PTGS2, IL6, CXCL8, and JUN) of HLJDD in treating RA were identified through constructing the PPI network of the 41 common targets from HLJDD and RA. These targets were significantly enriched in the biological process of the cellular inflammatory response to different substances such as organic substance and chemical and cytokine stimulus and associated with IL-17 signaling pathway (CCL2, CXCL10, CXCL8, IFN-γ, IL1β, IL6, JUN, MMP9, PTGS2), TNF signaling pathway (CCL2, CXCL10, ICAM1, IL1β, IL6, JUN, MMP9, PTGS2), rheumatoid arthritis pathway (CCL2, CXCL8, ICAM1, IFNG, IL1β, IL6, JUN), and Toll-like receptor signaling pathway (CXCL10, CXCL8, IL1β, IL6, JUN). Accumulating studies showed that IL-17 and IL-17-producing T helper (Th17) cells played a critical role during the development and progression of RA (Kim et al., 2017) and Lee’s study exhibited that IL-17 upregulated the expression of TLR3 by STAT3 pathways in fibroblast-like synoviocytes of RA (Lee et al., 2014). Tumor necrosis factor (TNF) signaling pathway has been reported to mediate leukocyte recruitment and inflammatory responses, and various anti-TNF inhibitors also acquired prominent potent effects on RA (Yang et al., 2018). In addition, Toll-like receptor (TLRs) and its corresponding downstream signaling pathways, such as Wnt, NF-κB, and MAPK signaling pathways, have been demonstrated in the biological process of synovial inflammation and bone remodeling of RA (Andreakos et al., 2005). Interestingly, low IL-10 and IFN-γ levels were found in RA, and this contradictory finding might be explained by published studies. Seung et al. (Lee et al., 2017) found IFN-γ regulated inflammatory cell death by targeting necroptosis, and IFN-γ deficiency increased the number of Th17 cells and upregulated the expression levels of IL-17 and TNF-α using experimental mice of collagen-induced arthritis (CIA). In addition, an authoritative study declared that IL-10 was also considered a highly promising treatment target for RA attributing to its unique capacity to inhibit cellular immunity and deactivate macrophages through downregulating the production of multiple proinflammatory cytokines, including IL1 and TNF-α (St Clair, 1999). Overall, the results suggested that complicated immune-associated signaling pathways were activated in the progression of RA, and inhibiting associated targets would alleviate the disease activity and prognosis for RA.

As important inflammatory regulators, IL6 and IL1β belong to the family of cytokines and could stimulate the production of neutrophils and megakaryocytes, guide immune cell differentiation, and upregulate the Th17/Treg balance, which was pathologically associated with the development of chronic inflammatory diseases (Tanaka et al., 2018; Gomes da Silva et al., 2020). In addition, the clinical practice had authenticated that the therapeutic monoclonal antibody against IL6, such as tozumab, could acquire significant curative effects in the treatment of RA (Humby et al., 2021). The activity of macrophages in the synovial compartment was regulated by substantial inflammatory factors, especially chemokines families, which included four groups: the C, CC, CXC, and CX3C chemokine receptor families (Zlotnik and Yoshie, 2000). Interestingly, three hub targets belong to the CC (CCL2) and CXC (CXCL8, CXCL10) subfamilies, of which CXCL8 was produced constitutively by macrophages in the synovial tissues and demonstrated to induce synovial inflammation in the animal model (Endo et al., 1994), and the main function of CCL2 was responsible for the recruitment of macrophages (Hachicha et al., 1995). Interferon-gamma (IFN-γ) was predominantly produced by T lymphocytes and natural killer (NK) cells and played an essential role in autoimmune disorders through activating multiple immune-related pathways, especially the JAK-STAT pathway (Simon et al., 2021). In Masaru’s study, a high level of IFN-γ was found associated with the severity and poor prognosis of RA, and the expression level of IFN-γ was significantly decreased after the treatment of JAK inhibitors (Kato, 2020). Matrix metalloproteinase-9 (MMP9) played a critical role in degrading components of extracellular matrix (ECM) and leukocyte migration in rheumatoid arthritis patients (Alwan and Ghali, 2021). Prostaglandin-endoperoxide synthase 2 (PTGS2), also called COX-2, has been recognized as a significant proinflammatory target of RA, and COX-2 inhibitor (NSAIDs) was the preferred anti-inflammatory drug to control pain and stiffness (Crofford, 2013). Notably, Nicole et al. found that the AP-1 Transcription Factor (JUN) could directly activate proinflammatory factor COX-2 in macrophages to promote arthritis in rat models (Hannemann et al., 2017). Summarily, the potential mechanisms of HLJDD in the treatment of RA might be attributed to the following aspects: inhibiting the immune inflammatory response, reducing the release of chemokines, and alleviating the destruction of ECM in the synovial compartment.

However, there still are some limitations in our study. On the one hand, although network pharmacology provides a comprehensive approach based on the combination of pharmacology, biochemics, bioinformatics, and network biology, the data of active ingredients and targets were collectively obtained from multi-public databases, and still selection bias exists. On the other hand, in this study, we just validated quercetin’s therapeutic role in inhibiting the proliferation of RA cells *in vitro*, but the ingredients-targets network and concrete molecular mechanism of HLJDD in the treatment of RA remain to be verified by in-depth *in vivo* and *in vitro* studies. Finally, the curative effect of quercetin might be non-specific due to artificial synthesis in this experiment and need more validation using extractive compounds in the future.

## Conclusion

In this study, we successfully screened and obtained the pivotal ingredients of HLJDD in treating RA, and 10 hub targets were identified to construct the botanical drugs-ingredients-targets-disease network. Functional enrichment analysis suggested HLJDD could regulate related targets through associated pathways, including TNF signaling pathway, Toll-like receptor signaling pathway, and IL-17 signaling pathway. Furthermore, based on the molecular docking analysis, important compounds such as quercetin, beta-sitosterol, wogonin, kaempferol, coptisine, and stigmasterol exhibited good binding ability with these core proteins. In conclusion, our study systematically expounded the mechanisms and molecular targets of HLJDD in the treatment of RA through the network pharmacology and molecular docking approach. The therapeutic action of HLJDD in RA was mainly by inhibiting the immune inflammatory response, reducing the release of chemokines, and alleviating the destruction of ECM in the synovial compartment.

## Data Availability

The original contributions presented in the study are included in the article/Supplementary Material, further inquiries can be directed to the corresponding authors.

## References

[B1] Abbasi M. Mousavi M. J. Jamalzehi S. Alimohammadi R. Bezvan M. H. Mohammadi H. (2019). Strategies toward Rheumatoid Arthritis Therapy; the Old and the New. J. Cel Physiol 234, 10018–10031. 10.1002/jcp.27860 30536757

[B2] Alwan I. T. Ghali K. H. (2021). Association Risk of Metalomatrix Proteinase Enzymes Levels (MMP-1, MMP-9 and MMP-13) with Development of Rheumatoid Arthritis. Ann. Rom. Soc. Cel Biol. 25, 11369–11378.

[B3] Andreakos E. Sacre S. Foxwell B. M. Feldmann M. (2005). The Toll-like Receptor-Nuclear Factor kB Pathway in Rheumatoid Arthritis. Front. Biosci. 10, 2478–2488. 10.2741/1712 15970510

[B4] Bie B. Sun J. Guo Y. Li J. Jiang W. Yang J. (2017). Baicalein: A Review of its Anti-cancer Effects and Mechanisms in Hepatocellular Carcinoma. Biomed. Pharmacother. 93, 1285–1291. 10.1016/j.biopha.2017.07.068 28747003

[B5] Bindea G. Galon J. Mlecnik B. (2013). CluePedia Cytoscape Plugin: Pathway Insights Using Integrated Experimental and In Silico Data. Bioinformatics 29, 661–663. 10.1093/bioinformatics/btt019 23325622 PMC3582273

[B6] Bindea G. Mlecnik B. Hackl H. Charoentong P. Tosolini M. Kirilovsky A. (2009). ClueGO: a Cytoscape Plug-In to Decipher Functionally Grouped Gene Ontology and Pathway Annotation Networks. Bioinformatics 25, 1091–1093. 10.1093/bioinformatics/btp101 19237447 PMC2666812

[B7] Burley S. K. Bhikadiya C. Bi C. Bittrich S. Chen L. Crichlow G. V. (2021). RCSB Protein Data Bank: Powerful New Tools for Exploring 3D Structures of Biological Macromolecules for Basic and Applied Research and Education in Fundamental Biology, Biomedicine, Biotechnology, Bioengineering and Energy Sciences. Nucleic Acids Res. 49, D437–D451. 10.1093/nar/gkaa1038 33211854 PMC7779003

[B8] Chen S.-J. Lin G.-J. Chen J.-W. Wang K.-C. Tien C.-H. Hu C.-F. (2019). Immunopathogenic Mechanisms and Novel Immune-Modulated Therapies in Rheumatoid Arthritis. Ijms 20, 1332. 10.3390/ijms20061332 PMC6470801 30884802

[B9] Chin C.-H. Chen S.-H. Wu H.-H. Ho C.-W. Ko M.-T. Lin C.-Y. (2014). cytoHubba: Identifying Hub Objects and Sub-networks from Complex Interactome. BMC Syst. Biol. 8, S11. 10.1186/1752-0509-8-S4-S11 25521941 PMC4290687

[B10] Conigliaro P. Triggianese P. De Martino E. Fonti G. L. Chimenti M. S. Sunzini F. (2019). Challenges in the Treatment of Rheumatoid Arthritis. Autoimmun. Rev. 18, 706–713. 10.1016/j.autrev.2019.05.007 31059844

[B11] Crofford L. J. (2013). Use of NSAIDs in Treating Patients with Arthritis. Arthritis Res. Ther. 15, S2. 10.1186/ar4174 PMC3891482 24267197

[B12] Deng J.-L. Xu Y.-h. Wang G. (2019). Identification of Potential Crucial Genes and Key Pathways in Breast Cancer Using Bioinformatic Analysis. Front. Genet. 10, 695. 10.3389/fgene.2019.00695 31428132 PMC6688090

[B13] Endo H. Akahoshi T. Nishimura A. Tonegawa M. Takagishi K. Kashiwazaki S. (1994). Experimental Arthritis Induced by Continuous Infusion of IL-8 into Rabbit Knee Joints. Clin. Exp. Immunol. 96, 31–35. 10.1111/j.1365-2249.1994.tb06225.x 7512008 PMC1534528

[B14] Fang Q. Zhan X. P. Mo J. L. Sun M. (2004). The Effect of Huanglian Jiedu Tang on Alzheimer's Disease and its Influence on Cytokines. Zhongguo Zhong Yao Za Zhi 29, 575–578. 15706928

[B15] Gomes da Silva I. I. F. Lima C. A. D. Monteiro M. L. A. Barboza D. A. S. P. Rushansky E. Mariano M. H. Q. d. A. (2020). IL1β, IL18, NFKB1 and IFNG Gene Interactions Are Associated with Severity of Rheumatoid Arthritis: A Pilot Study. Autoimmunity 53, 95–101. 10.1080/08916934.2019.1710831 31992083

[B16] Gu X. R. Fang S. Y. Ren W. Wang H. J. Yang J. Si N. (2018). Pharmacodynamics of Huanglian Jiedu Decoction in Alzheimer's Disease (AD) Model Rats and Effect on Improvement of Inflammation Microenvironment in Brain. Zhongguo Zhong Yao Za Zhi 43, 3006–3011. 10.19540/j.cnki.cjcmm.2018.0092 30111062

[B17] Hachicha M. Naccache P. H. McColl S. R. (1995). Inflammatory Microcrystals Differentially Regulate the Secretion of Macrophage Inflammatory Protein 1 and Interleukin 8 by Human Neutrophils: a Possible Mechanism of Neutrophil Recruitment to Sites of Inflammation in Synovitis. J. Exp. Med. 182, 2019–2025. 10.1084/jem.182.6.2019 7500047 PMC2192242

[B18] Hannemann N. Jordan J. Paul S. Reid S. Baenkler H.-W. Sonnewald S. (2017). The AP-1 Transcription Factor C-Jun Promotes Arthritis by Regulating Cyclooxygenase-2 and Arginase-1 Expression in Macrophages. J.I. 198, 3605–3614. 10.4049/jimmunol.1601330 28298526

[B19] Hu Y. Hu Z. Wang S. Dong X. Xiao C. Jiang M. (2013). Protective Effects of Huang-Lian-Jie-Du-Tang and its Component Group on Collagen-Induced Arthritis in Rats. J. Ethnopharmacology 150, 1137–1144. 10.1016/j.jep.2013.10.038 24212076

[B20] Huang L. Lv Q. Xie D. Shi T. Wen C. (2016). Deciphering the Potential Pharmaceutical Mechanism of Chinese Traditional Medicine (Gui-Zhi-Shao-Yao-Zhi-Mu) on Rheumatoid Arthritis. Sci. Rep. 6, 22602. 10.1038/srep22602 26935797 PMC4776278

[B21] Humby F. Durez P. Buch M. H. Lewis M. J. Rizvi H. Rivellese F. (2021). Rituximab versus Tocilizumab in Anti-TNF Inadequate Responder Patients with Rheumatoid Arthritis (R4RA): 16-week Outcomes of a Stratified, Biopsy-Driven, Multicentre, Open-Label, Phase 4 Randomised Controlled Trial. The Lancet 397, 305–317. 10.1016/S0140-6736(20)32341-2 PMC7829614 33485455

[B22] Kato M. (2020). New Insights into IFN-γ in Rheumatoid Arthritis: Role in the Era of JAK Inhibitors. Immunological Med. 43, 72–78. 10.1080/25785826.2020.1751908 32338187

[B23] Khan N. M. Haseeb A. Ansari M. Y. Devarapalli P. Haynie S. Haqqi T. M. (2017). Wogonin, a Plant Derived Small Molecule, Exerts Potent Anti-inflammatory and Chondroprotective Effects through the Activation of ROS/ERK/Nrf2 Signaling Pathways in Human Osteoarthritis Chondrocytes. Free Radic. Biol. Med. 106, 288–301. 10.1016/j.freeradbiomed.2017.02.041 28237856 PMC5490997

[B24] Kim E. K. Kwon J.-E. Lee S.-Y. Lee E.-J. Kim D. S. Moon S.-J. (2017). IL-17-mediated Mitochondrial Dysfunction Impairs Apoptosis in Rheumatoid Arthritis Synovial Fibroblasts through Activation of Autophagy. Cell Death Dis 8, e2565. 10.1038/cddis.2016.490 PMC5386390 28102843

[B25] Lee S.-Y. Yoon B.-Y. Kim J.-I. Heo Y.-M. Woo Y.-J. Park S.-H. (2014). Interleukin-17 Increases the Expression of Toll-like Receptor 3 via the STAT3 Pathway in Rheumatoid Arthritis Fibroblast-like Synoviocytes. Immunology 141, 353–361. 10.1111/imm.12196 24708416 PMC3930374

[B26] Lee S. H. Kwon J. y. Kim S.-Y. Jung K. Cho M.-L. (2017). Interferon-gamma Regulates Inflammatory Cell Death by Targeting Necroptosis in Experimental Autoimmune Arthritis. Sci. Rep. 7, 10133. 10.1038/s41598-017-09767-0 28860618 PMC5579272

[B27] Li H. Liu L. Liu C. Zhuang J. Zhou C. Yang J. (2018a). Deciphering Key Pharmacological Pathways of Qingdai Acting on Chronic Myeloid Leukemia Using a Network Pharmacology-Based Strategy. Med. Sci. Monit. 24, 5668–5688. 10.12659/MSM.908756 30108199 PMC6106618

[B28] Li X. Tang H. Tang Q. Chen W. (2021). Decoding the Mechanism of Huanglian Jiedu Decoction in Treating Pneumonia Based on Network Pharmacology and Molecular Docking. Front. Cel Dev. Biol. 9, 638366. 10.3389/fcell.2021.638366 PMC7930397 33681222

[B29] Li Y. H. Yu C. Y. Li X. X. Zhang P. Tang J. Yang Q. (2018b). Therapeutic Target Database Update 2018: Enriched Resource for Facilitating Bench-To-Clinic Research of Targeted Therapeutics. Nucleic Acids Res. 46, D1121–D1127. 10.1093/nar/gkx1076 29140520 PMC5753365

[B30] Li Y. Yao J. Han C. Yang J. Chaudhry M. Wang S. (2016). Quercetin, Inflammation and Immunity. Nutrients 8, 167. 10.3390/nu8030167 26999194 PMC4808895

[B31] Lin M.-K. Yu Y.-L. Chen K.-C. Chang W.-T. Lee M.-S. Yang M.-J. (2011). Kaempferol from Semen Cuscutae Attenuates the Immune Function of Dendritic Cells. Immunobiology 216, 1103–1109. 10.1016/j.imbio.2011.05.002 21621872

[B32] Liu W. Zeng Y. Li Y. Li N. Peng M. Cheng J. (2021). Exploring the Potential Targets and Mechanisms of Huang Lian Jie Du Decoction in the Treatment of Coronavirus Disease 2019 Based on Network Pharmacology. Int. J. Gen. Med. 14, 9873–9885. 10.2147/IJGM.S337025 34938107 PMC8687521

[B33] Liu Z. Guo F. Wang Y. Li C. Zhang X. Li H. (2016). BATMAN-TCM: a Bioinformatics Analysis Tool for Molecular mechANism of Traditional Chinese Medicine. Sci. Rep. 6, 21146. 10.1038/srep21146 26879404 PMC4754750

[B34] Lu J. Wang J.-S. Kong L.-Y. (2011). Anti-inflammatory Effects of Huang-Lian-Jie-Du Decoction, its Two Fractions and Four Typical Compounds. J. Ethnopharmacology 134, 911–918. 10.1016/j.jep.2011.01.049 21296144

[B35] Lu J. Wang Z. Li S. Xin Q. Yuan M. Li H. (2018). Quercetin Inhibits the Migration and Invasion of HCCLM3 Cells by Suppressing the Expression of P-Akt1, Matrix Metalloproteinase (MMP) MMP-2, and MMP-9. Med. Sci. Monit. 24, 2583–2589. 10.12659/MSM.906172 29701200 PMC5941983

[B36] Luo J. Li H. Liu Z. Li C. Wang R. Fang J. (2020). Integrative Analyses of Gene Expression Profile Reveal Potential Crucial Roles of Mitotic Cell Cycle and Microtubule Cytoskeleton in Pulmonary Artery Hypertension. BMC Med. Genomics 13, 86. 10.1186/s12920-020-00740-x 32586319 PMC7318763

[B37] Qu S.-Y. Li X.-Y. Heng X. Qi Y.-Y. Ge P.-Y. Ni S.-j. (2021). Analysis of Antidepressant Activity of Huang-Lian Jie-Du Decoction through Network Pharmacology and Metabolomics. Front. Pharmacol. 12, 619288. 10.3389/fphar.2021.619288 33746756 PMC7970346

[B38] Raudvere U. Kolberg L. Kuzmin I. Arak T. Adler P. Peterson H. (2019). g:Profiler: a Web Server for Functional Enrichment Analysis and Conversions of Gene Lists (2019 Update). Nucleic Acids Res. 47, W191–W198. 10.1093/nar/gkz369 31066453 PMC6602461

[B39] Rigden D. J. Fernández X. M. (2020). The 27th Annual Nucleic Acids Research Database Issue and Molecular Biology Database Collection. Nucleic Acids Res. 48, D1–D8. 10.1093/nar/gkz1161 31906604 PMC6943072

[B40] Ru J. Li P. Wang J. Zhou W. Li B. Huang C. (2014). TCMSP: a Database of Systems Pharmacology for Drug Discovery from Herbal Medicines. J. Cheminform 6, 13. 10.1186/1758-2946-6-13 24735618 PMC4001360

[B41] Seeliger D. de Groot B. L. (2010). Ligand Docking and Binding Site Analysis with PyMOL and Autodock/Vina. J. Comput. Aided Mol. Des. 24, 417–422. 10.1007/s10822-010-9352-6 20401516 PMC2881210

[B42] Shannon P. Markiel A. Ozier O. Baliga N. S. Wang J. T. Ramage D. (2003). Cytoscape: a Software Environment for Integrated Models of Biomolecular Interaction Networks. Genome Res. 13, 2498–2504. 10.1101/gr.1239303 14597658 PMC403769

[B43] Simon L. S. Taylor P. C. Choy E. H. Sebba A. Quebe A. Knopp K. L. (2021). The Jak/STAT Pathway: A Focus on Pain in Rheumatoid Arthritis. Semin. Arthritis Rheum. 51, 278–284. 10.1016/j.semarthrit.2020.10.008 33412435

[B44] Smolen J. S. Aletaha D. McInnes I. B. (2016). Rheumatoid Arthritis. The Lancet 388, 2023–2038. 10.1016/S0140-6736(16)30173-8 27156434

[B45] Song W. Ni S. Fu Y. Wang Y. (2018). Uncovering the Mechanism of Maxing Ganshi Decoction on Asthma from a Systematic Perspective: A Network Pharmacology Study. Sci. Rep. 8, 17362. 10.1038/s41598-018-35791-9 30478434 PMC6255815

[B46] St Clair E. W. (1999). Interleukin 10 Treatment for Rheumatoid Arthritis. Ann. Rheum. Dis. 58 (Suppl. 1), I99–I102. 10.1136/ard.58.2008.i99 10577984 PMC1766579

[B47] Sung M.-S. Lee E.-G. Jeon H.-S. Chae H.-J. Park S. J. Lee Y. C. (2012). Quercetin Inhibits IL-1β-Induced Proliferation and Production of MMPs, COX-2, and PGE2 by Rheumatoid Synovial Fibroblast. Inflammation 35, 1585–1594. 10.1007/s10753-012-9473-2 22592909

[B48] Szklarczyk D. Gable A. L. Lyon D. Junge A. Wyder S. Huerta-Cepas J. (2019). STRING V11: Protein-Protein Association Networks with Increased Coverage, Supporting Functional Discovery in Genome-wide Experimental Datasets. Nucleic Acids Res. 47, D607–D613. 10.1093/nar/gky1131 30476243 PMC6323986

[B49] Tanaka T. Narazaki M. Kishimoto T. (2018). Interleukin (IL-6) Immunotherapy. Cold Spring Harb Perspect. Biol. 10, a028456. 10.1101/cshperspect.a028456 28778870 PMC6071487

[B50] UniProt Consortium (2019). UniProt: a Worldwide Hub of Protein Knowledge. Nucleic Acids Res. 47, D506–D515. 10.1093/nar/gky1049 30395287 PMC6323992

[B51] Wang C. Qu Z. Kong L. Xu L. Zhang M. Liu J. (2019). Quercetin Ameliorates Lipopolysaccharide-Caused Inflammatory Damage via Down-Regulation of miR-221 in WI-38 Cells. Exp. Mol. Pathol. 108, 1–8. 10.1016/j.yexmp.2019.03.002 30849307

[B52] Wang Y. Bryant S. H. Cheng T. Wang J. Gindulyte A. Shoemaker B. A. (2017). PubChem BioAssay: 2017 Update. Nucleic Acids Res. 45, D955–D963. 10.1093/nar/gkw1118 27899599 PMC5210581

[B53] Wei S. Mingquan W. U. Wang H. Huang Y. Zhou H. Zhang L. (2016). Network Pharmacology Study on Huanglianjiedu Decoction in Treatment of Hepatitis and Liver Fibrosis. Evaluation and Analysis of Drug-Use in Hospitals of China 16 (10), 1308–1310. 10.14009/j.issn.1672-2124.2016.10.004

[B54] Whirl-Carrillo M. McDonagh E. M. Hebert J. M. Gong L. Sangkuhl K. Thorn C. F. (2012). Pharmacogenomics Knowledge for Personalized Medicine. Clin. Pharmacol. Ther. 92, 414–417. 10.1038/clpt.2012.96 22992668 PMC3660037

[B55] Wilsdon T. D. Hill C. L. (2017). Managing the Drug Treatment of Rheumatoid Arthritis. Aust. Prescr 40, 51–58. 10.18773/austprescr.2017.012 28507397 PMC5408004

[B56] Wishart D. S. Feunang Y. D. Guo A. C. Lo E. J. Marcu A. Grant J. R. (2018). DrugBank 5.0: a Major Update to the DrugBank Database for 2018. Nucleic Acids Res. 46, D1074–D1082. 10.1093/nar/gkx1037 29126136 PMC5753335

[B57] Yan W. Ma X. Zhao X. Zhang S. (2018). Baicalein Induces Apoptosis and Autophagy of Breast Cancer Cells via Inhibiting PI3K/AKT Pathway *In Vivo* and Vitro. Dddt 12, 3961–3972. 10.2147/DDDT.S181939 30510404 PMC6248272

[B58] Yang S. Wang J. Brand D. D. Zheng S. G. (2018). Role of TNF-TNF Receptor 2 Signal in Regulatory T Cells and its Therapeutic Implications. Front. Immunol. 9, 784. 10.3389/fimmu.2018.00784 29725328 PMC5916970

[B59] Yang Y. Wang H.-J. Yang J. Brantner A. H. Lower-Nedza A. D. Si N. (2013). Chemical Profiling and Quantification of Chinese Medicinal Formula Huang-Lian-Jie-Du Decoction, a Systematic Quality Control Strategy Using Ultra High Performance Liquid Chromatography Combined with Hybrid Quadrupole-Orbitrap and Triple Quadrupole Mass Spectrometers. J. Chromatogr. A 1321, 88–99. 10.1016/j.chroma.2013.10.072 24231264

[B60] Yuan Z. Yang L. Zhang X. Ji P. Hua Y. Wei Y. (2019). Huang-Lian-Jie-Du Decoction Ameliorates Acute Ulcerative Colitis in Mice via Regulating NF-Κb and Nrf2 Signaling Pathways and Enhancing Intestinal Barrier Function. Front. Pharmacol. 10, 1354. 10.3389/fphar.2019.01354 31849642 PMC6900672

[B61] Zlotnik A. Yoshie O. (2000). Chemokines. Immunity 12, 121–127. 10.1016/s1074-7613(00)80165-x 10714678

