# Supplementary figures and images for: A Preliminary Inquiry Into the Potential Mechanism of Huang-Lian-Jie-Du Decoction in Treating Rheumatoid Arthritis via Network Pharmacology and Molecular Docking
Source: Front Cell Dev Biol. 2022 Jan 19;9:740266. doi: 10.3389/fcell.2021.740266 (PMC8807552; doi:10.3389/fcell.2021.740266)

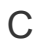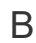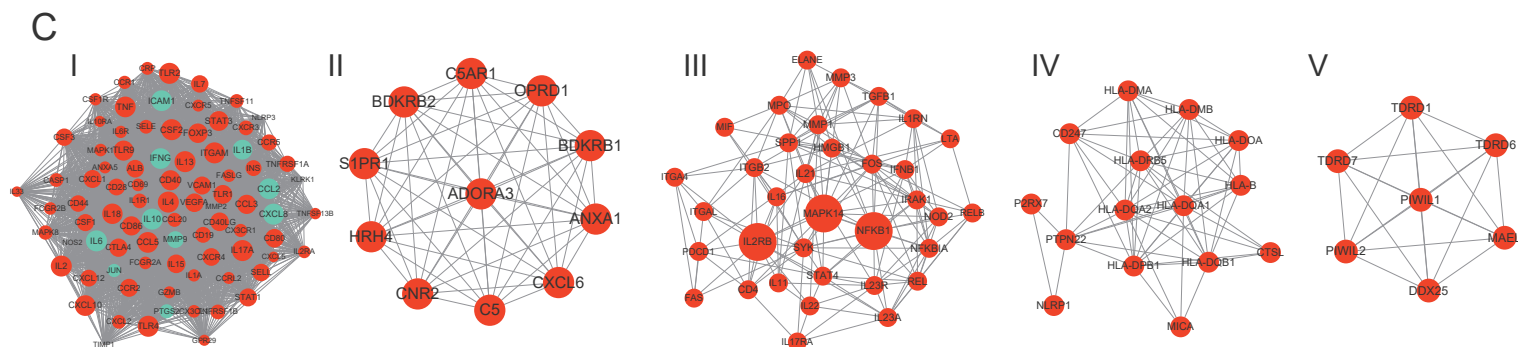

Supplement: Supplementary file 3 [file DataSheet1.PDF]
